# An anterolateral thigh chimeric flap for dynamic facial and esthetic reconstruction after oncological surgery in the maxillofacial region: a case report

**DOI:** 10.1186/s13005-018-0164-6

**Published:** 2018-04-11

**Authors:** Zoltán Lóderer, Tamás Vereb, Róbert Paczona, Ágnes Janovszky, József Piffkó

**Affiliations:** Department of Oral and Maxillofacial Surgery, University of Szeged, Kálvária 57, Szeged, H-6725 Hungary

**Keywords:** Maxillofacial surgery, Microsurgery, Dynamic reconstruction, Basal cell carcinoma, Anterolateral thigh flap

## Abstract

**Background:**

The surgical management of malignant tumors in the head and neck region often leads to functional and esthetic defects that impair the quality of life of the patients. Reconstruction can be solved with prostheses in these cases, but various types of microsurgical free flaps can provide a better clinical outcome.

**Case presentation:**

In this case report, the tumor and parts of the involved facial muscles and nerve were excised surgically from a 42-year-old patient after a third relapse of basal cell carcinoma in the left midface. The tissue defect was reconstructed with an anterolateral thigh chimeric type I fascio-myocutaneous flap, where the facial palsy was restored with a segmental branch of the femoral nerve and the involved mouth corner elevator muscles for the segmented vastus lateralis muscle. The 6-month follow-up revealed a good esthetic outcome, the soft tissue defect reconstruction with good functional activity of the reconstructed facial nerve and with acceptable mimic movements. There has been no subsequent recurrence.

**Conclusions:**

It is concluded that the chimeric type I anterolateral fascio-myocutaneous free flap can offer a good option for the esthetic and functional reconstruction of an extensive tissue defect in the maxillofacial region.

## Background

Surgical resection of a malignant tumor in the head and neck region as part of complex oncological management has a considerable impact on the clinical outcome. These procedures often result in severe defects not only in the craniofacial bones, but also in the soft tissue coverage and function of the mimetic muscles, and the complexity of these lesions necessitates the use of different types of tissues with different functions for the reconstruction.

The recovery of maxillofacial integrity can be achieved by means of free or vascularized autologous bone transplantation, but allogeneic bone or artificial materials also play an important role in the reconstruction of maxillofacial defects. The soft tissue coverage is a basic part of the primary treatment. While local and regional flaps offer a simple practicable solution, free flaps (currently applied in an increasing number) can provide significantly more satisfactory esthetic and functional results. A common complication after oncological surgery in the maxillofacial region is partial or total facial palsy, which leads to the development of problems such as the inability to elevate the eyebrow, caused by the temporal branch lesion with consequent frontal muscle palsy, and the eye dryness or epiphora induced by the deficiency of the orbicularis oculi muscle [1]. The lack of other oral functions and their consequences (e.g. the inability to elevate the corner of the mouth, and drooling) can have marked effect on social interactions [2, 3]. Many factors are involved in the selection of an appropriate flap, such as the size and location of the defect, the blood supply of the flap and recipient site, or the aim of the reconstructive intervention (e.g. esthetic or functional expectations). An awareness of the functional anatomical aspects and high-level experience in microsurgical reconstruction are therefore essential if the patient is to achieve an acceptable aesthetic or functional outcome.

## Case presentation

**Fig. 1:**
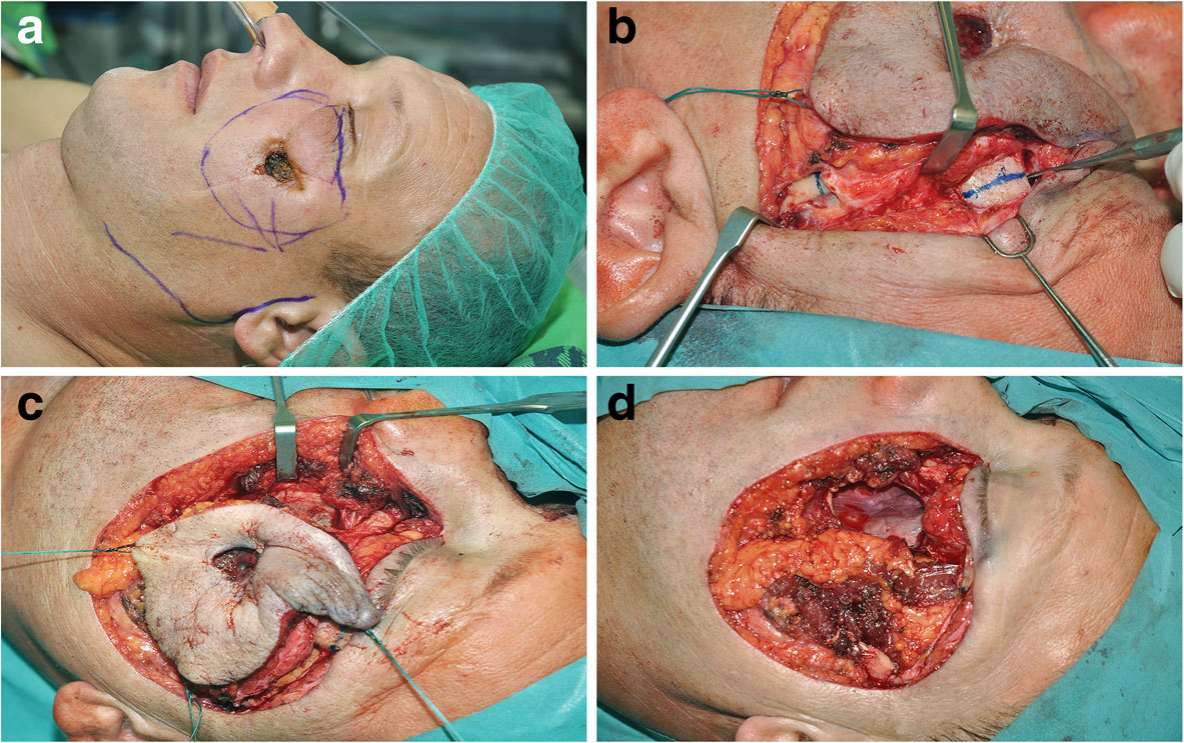
Preoperative appearance of the basal cell carcinoma in the left midface (**a**) and the radical tumor resection procedure (**b-d**)

**Fig. 2:**
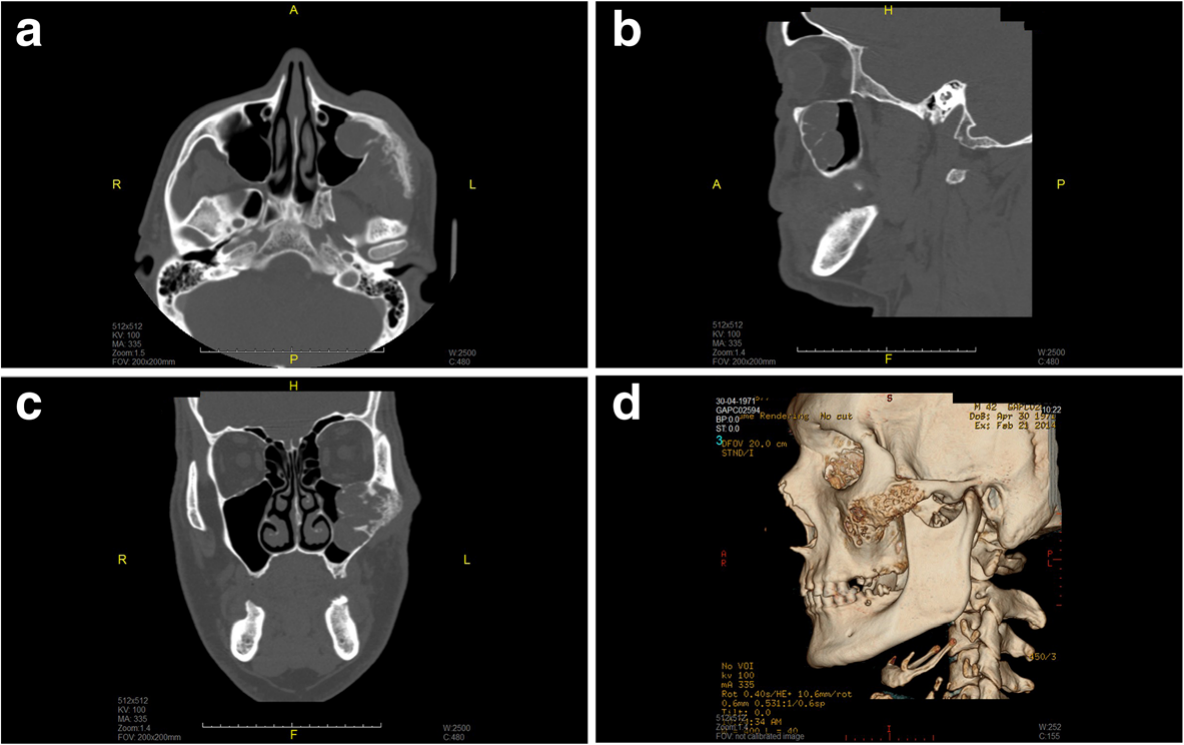
CT scans in coronal (**a**), sagittal (**b**) and axial (**c**) views and 3-dimensional reconstruction in a lateral view (**d**)

A third relapse of basal cell carcinoma was confirmed by histology in the left midfacial region of a 42-year-old patient (Fig 1A). The preoperative CT imaging demonstrated that the tumor invaded the maxillary sinus, the orbital floor and the surrounding soft tissues, e.g. facial skin, the subcutaneous tissue and the mouth elevator muscles (Fig 2).

Radical tumor resection combined with partial maxillectomy and wide peritumoral soft tissue resection was performed. The marginal mandibular branch of the facial nerve could be salvaged, but the zygomaticobuccal branches of the mimetic muscles were ablated due to their infiltration by the tumor. The zygomaticus major and minor, levator anguli oris, levator labii superioris and buccinator muscles were resected (Fig 1B-D). Following partial maxillectomy, the orbital floor was reconstructed with a titanium mesh (Titanium Contourable Mesh Plates, malleable, 1.3 mm, Synthes Medical Hungary, Budapest, Hungary).

**Fig. 3:**
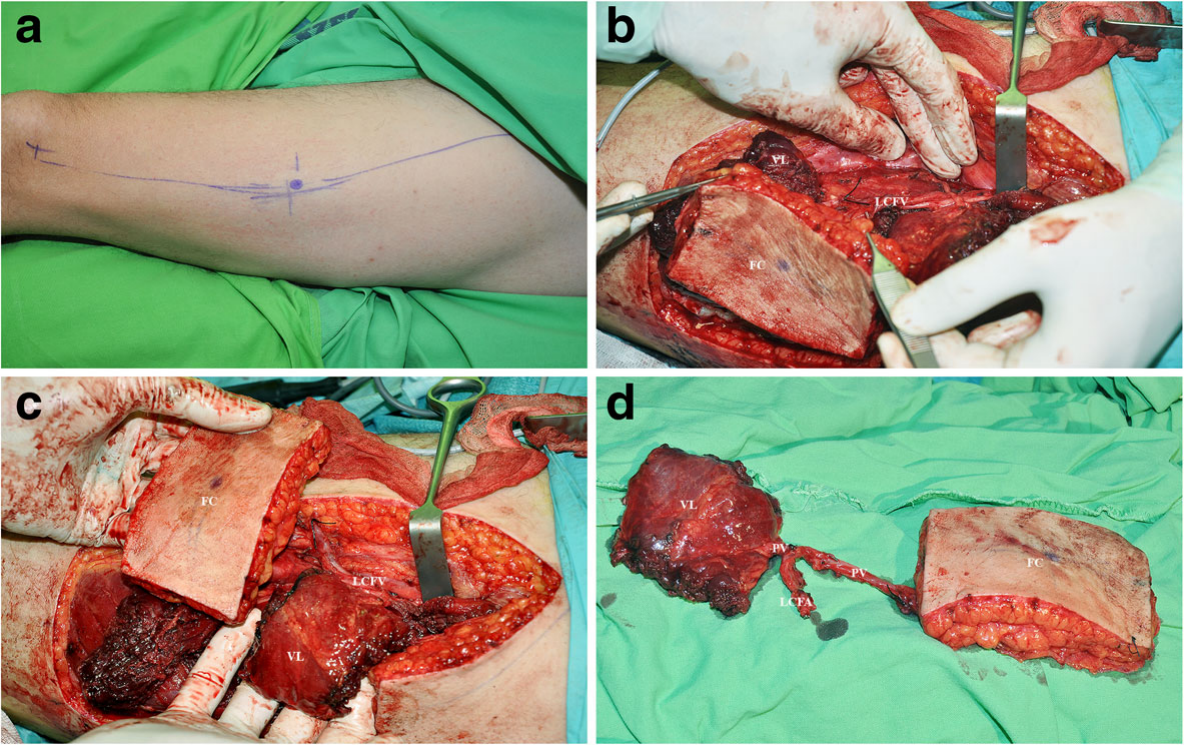
Marking of the surgical site and the perforator vessel on the left thigh (**a**). Raising of the chimeric type I anterolateral fasciocutaneous (FC) and vastus lateralis muscle segment (VL) flap with the circumflex femoral vessels (LCFV) (**b**, **c**), and the segmental branch of the femoral nerve and perforator vessels (PV) (**d**)

**Fig. 4:**
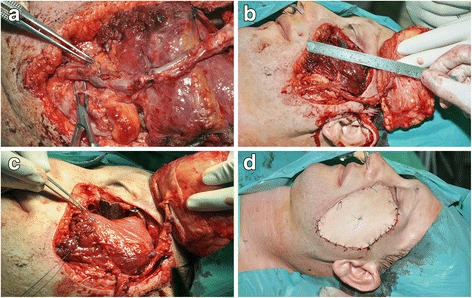
Blood vessel and nerve anastomoses on the recipient side (**a**), the flap position (**b**, **c**), and the state directly after the surgical reconstruction (**d**)

In parallel with the tumor resection, a chimeric type I ALT fasciocutaneous and a vastus lateralis muscle segment flap were harvested on the left thigh (Fig 3) [4]. Both were supplied by a perforator of the descending branch of the lateral circumflex femoral artery. The segmental branch of the femoral nerve innervating the selected muscle segment was identified by use of a bipolar electric stimulator (Aesculap GN015, B. Braun Melsungen AG, Melsungen, Germany) and was prepared under an operating microscope (HEZ 2429, Möller-Wedel GmbH & Co. KG, Wedel, Germany) by intraneural dissection in the nerve trunk in order to gain more nerve length. The vastus lateralis muscle was dissected and sectioned, providing an appropriate length with which to substitute the mouth corner elevators. The chimeric type I flap was transplanted onto the midfacial defect (Fig 4). The vastus lateralis muscle segment was fixed to the modiolus and temporal fascia with 2.0 monofilament, absorbable interrupted sutures (PDS®, Ethicon, One Johnson & Johnson Plaza, New Brunswick, New Jersey, USA). Vessel anastomoses were created between the left facial and left circumflex femoral arteries, and the left facial and left circumflex femoral veins. Dynamic functional reconstruction of the region was attempted by co-aptation of the motor nerve of the muscle and the previously selected buccal branch of the facial nerve.9.0 monofilament, non-absorbable (Prolene®, Ethicon, One Johnson & Johnson Plaza, New Brunswick, New Jersey, USA) interrupted sutures were applied to anastomose the above arteries and nerves, and two half running sutures in venous anastomosis. The perforator artery was signed with a stitch to allow observation of the perfusion with a hand-held Doppler probe. The recipient and donor site were closed primarily.

During the regular follow-up (monthly for 6 months), possible complications (such as bleeding, wound healing failure, or abscess formation) and functional improvements were checked.

**Fig. 5:**
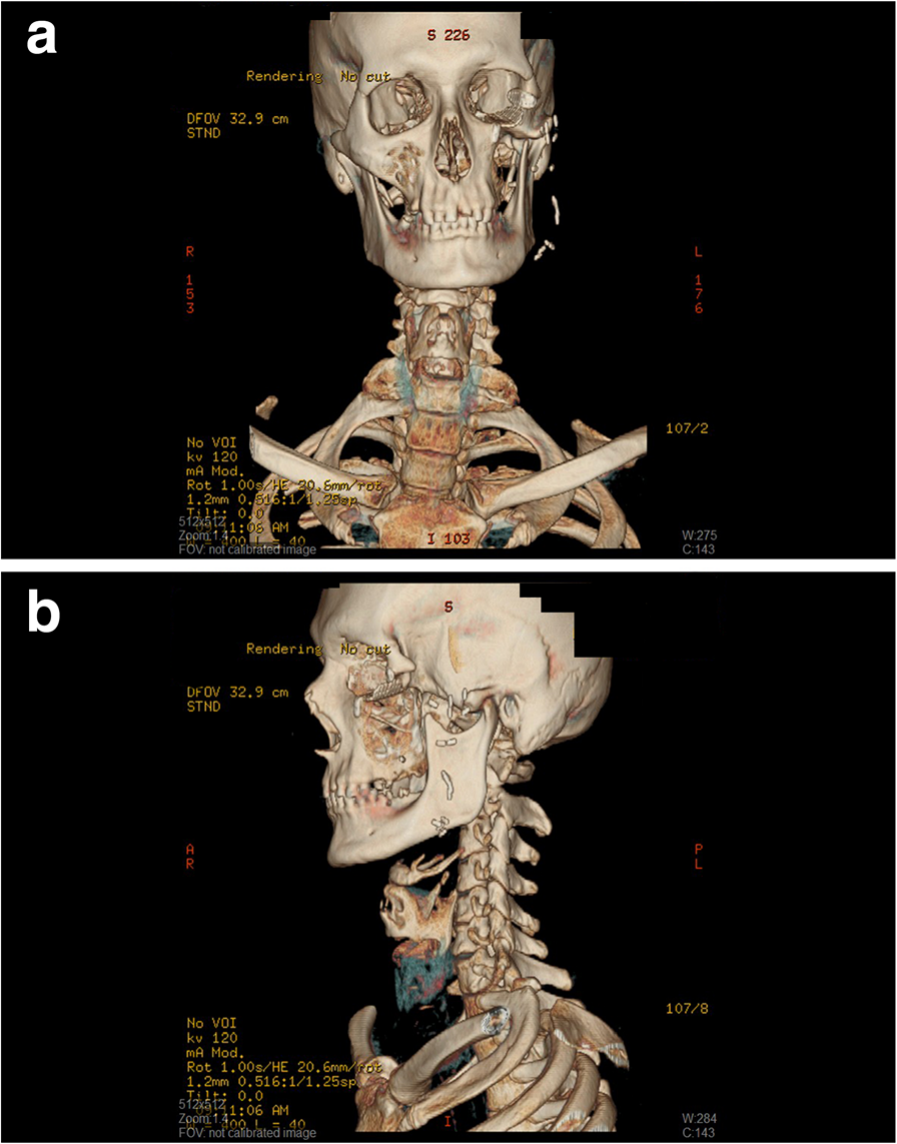
The tumor-free status revealed by CT 6 months after the operation in frontal (**a**) and lateral (**b**) view

Histological examination revealed basal cell carcinoma infiltrating the muscular and bony tissues and nest formation with palisaded tumor cells at the periphery. The histological sample indicated R0 resection with a wide tumor-free surgical margin. The subsequent CT imaging confirmed successful resection of the basal cell carcinoma with no tumor recurrence or pathological accumulation of contrast agent (Fig 5).

**Fig. 6:**
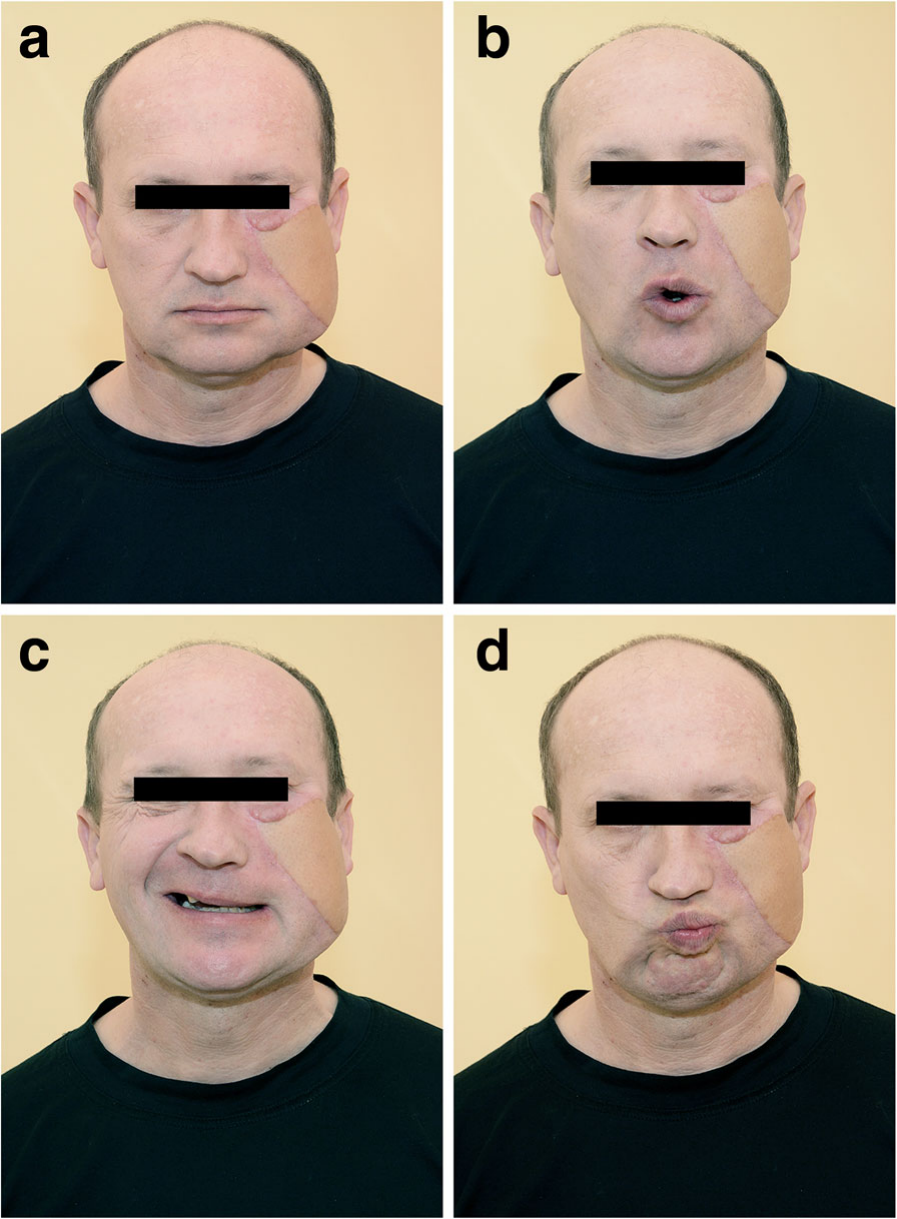
Esthetic appearance of the patient (**a**) and the function of the restored facial nerve 6 months after the surgical intervention (**b**-**d**)

A salivary fistula proceeding from the parotid gland was found and treated on an outpatient basis. Positional facial asymmetry was not observed in a standing or calm position. Certain functions of the mimetic muscle (such as lip rounding) returned, but ability to elevate the left corner of the mouth remained far below that on the intact side 6 months after the surgical management (Fig. 6).

## Discussion and conclusions

The literature provides only a limited number of recommendations as concerns the management of basal cell carcinoma with an extensive tissue defect. Various local flaps, such as a paramedian forehead flap, a lateral cheek rotation flap or a platysma myocutaneous flap, can be applied for the reconstruction of large maxillofacial defects after malignant lesion resection [5]. However, a study involving 685 patients with 765 basal cell carcinomas suggested that a better functional and esthetic result can be achieved through the use of pedicled flaps [6].

A common complication such as facial paralysis after maxillofacial surgery has a great impact on the social interaction of the patient. The aim of dynamic facial reconstruction is to achieve a symmetrical and coordinated smile, an enhanced cheek tone, improved speech and the ability to eat [7]. Coyle et al. published an algorithm for the therapeutic approaches to facial palsy at different stages after the neural impairment, but possible soft tissue defects were not considered [7]. Chuang discussed the therapeutic possibilities of long-standing facial paralysis, emphasizing the feasibility of regional muscle and microvascular free tissue transfer. While regional muscle transfer is reliable and provides the immediate return of movement without a spontaneous mimetic nature, it usually requires multiple surgery [8, 9]. Although these methods are often unable to restore full maxillofacial integrity and balance the facial movements, they are options for patients not eligible for free micro-neurovascular reconstruction [10, 11]. Free flaps can provide synchronous, mimetic movement, but a prolonged healing time may be required [8]. With regard to the extensive soft tissue defect after the surgical resection of the tumorous lesion and the general state of health of our patient, we applied an ALT chimeric flap to reconstruct soft tissue defect and to correct the facial paralysis. The blood supply of this flap is supported by the descending branch of the lateral circumflex femoral artery, its applicability therefore requiring a complex reconstructive solution [12].

The myocutaneous ALT flap can readily be obtained and may provide a good amount of muscle for filling of the tissue defect, together with the chance to reconstruct the bony defect in the craniofacial region. The thickness of the subcutaneous fat in the anterolateral area can be modified in order to achieve the necessary flap thickness, which makes it highly suitable for the surgical treatment of oral and maxillofacial defects [13, 14]. Donor site morbidity, such as reduced sensitivity around the scar, is a common complaint of the patients [15–18]. However, the donor site defect both esthetically and functionally in our case was minimal, and the quadriceps function was not affected.

The dynamic reconstruction of facial palsy demands careful patient selection and an appropriate surgical technique if excellent results are to be expected [7]. A number of studies have revealed that significantly better functional results are achieved if reconstruction surgery is performed within 2 years [19, 20]. Single-stage surgery (reconstruction of both the soft tissue defect and the facial palsy) may provide a better outcome, but the general state of health of a patient has to be considered and multistage operations may be unavoidable in certain cases. Various nerve grafts, such as those of the masseteric or segmental branch, influence the functional and aesthetic results. While the masseteric nerve guarantees free voluntary gracilis muscle activation without any spontaneous smiling, free flaps innervated by the segmental branch have a lower success rate and result in less movement; however, spontaneous smiling can be observed [21]. The age and expectations of our patient played an important role in the management of the maxillofacial integrity, including the decision concerning the microvascular free tissue transfer combined with the segmental nerve branch.

Oncological surgery in the head and neck region can often lead to complex functional and aesthetical defects. The management of these extensive impairments often involves therapeutic difficulties, and the surgeon may have to seek new opportunities to achieve acceptable results. In general, single-stage surgery is associated with fewer complications and better neural regeneration. The chimeric type I ALT flap can be a good option for facial dynamic reconstruction, but the surgeon must also consider individual anatomical variation and other potential therapeutic solutions with a view to obtaining a satisfactory clinical outcome.

## Abbreviations

ALT: anterolateral thigh
CT: computer tomography
